# Molecular Weight Cut-Off and Structural Analysis of Vacuum-Assisted Titania Membranes for Water Processing

**DOI:** 10.3390/ma9110938

**Published:** 2016-11-18

**Authors:** Siti Nurehan Abd Jalil, David K. Wang, Christelle Yacou, Julius Motuzas, Simon Smart, João C. Diniz da Costa

**Affiliations:** 1 The University of Queensland, FIM²Lab—Functional Interfacial Materials and Membranes Laboratory, School of Chemical Engineering, Brisbane 4072, Australia; s.abdjalil@uq.edu.au (S.N.A.J.); d.wang1@uq.edu.au (D.K.W.); Christelle.yacou@univ-ag.fr (C.Y.); j.motuzas@uq.edu.au (J.M.); s.smart@uq.edu.au (S.S.); 2 Faculty of Chemical Engineering, Universiti Teknologi MARA (UiTM), Shah Alam 40450, Malaysia; 3 Department of Engineering, Université des Antilles, BP 250, Pointe à Pitre Cedex 97157, France

**Keywords:** titania membranes, vacuum-assisted method, water flux, organic rejection

## Abstract

This work investigates the structural formation and analyses of titania membranes (TM) prepared using different vacuum exposure times for molecular weight (MW) cut-off performance and oil/water separation. Titania membranes were synthesized via a sol-gel method and coated on macroporous alumina tubes followed by exposure to a vacuum between 30 and 1200 s and then calcined at 400 °C. X-ray diffraction and nitrogen adsorption analyses showed that the crystallite size and particle size of titania increased as a function of vacuum time. All the TM membranes were mesoporous with an average pore diameter of ~3.6 nm with an anatase crystal morphology. Water, glucose, sucrose, and polyvinylpyrrolidone with 40 and 360 kDa (PVP-40 kDa and PVP-360 kDa) were used as feed solutions for MW cut-off and hexadecane solution for oil filtration investigation. The TM membranes were not able to separate glucose and sucrose, thus indicating the membrane pore sizes are larger than the kinetic diameter of sucrose of 0.9 nm, irrespective of vacuum exposure time. They also showed only moderate rejection (20%) of the smaller PVP-40 kDa, however, all the membranes were able to obtain an excellent rejection of near 100% for the larger PVP-360 kDa molecule. Furthermore, the TM membranes were tested for the separation of oil emulsions with a high concentration of oil (3000 ppm), reaching high oil rejections of more than 90% of oil. In general, the water fluxes increased with the vacuum exposure time indicating a pore structural tailoring effect. It is therefore proposed that a mechanism of pore size tailoring was formed by an interconnected network of Ti–O–Ti nanoparticles with inter-particle voids, which increased as TiO_2_ nanoparticle size increased as a function of vacuum exposure time, and thus reduced the water transport resistance through the TM membranes.

## 1. Introduction

Titania membranes are increasingly finding usability in water processing applications. Titania membranes can operate as membrane contactors to separate water from other substances, or as membrane reactors to take advantage of the catalytic properties of titania crystals to destroy organic compounds whilst separating water. The application of titania membranes includes processing textile wastewaters [1], desalination [2], microfiltration of pectin solutions [3], and oil/water separation [4], among many other examples. The photo-catalytic properties of titania membranes via the active anatase [5] phase have been reported for the degradation of phenol [6], azo-dyes [7], and for the treatment of domestic wastewater [8]. Titania membranes were also reported to have a dual-function for wastewater processing using ozonation and water separation [9]. The control of the pore sizes of the titania membranes is therefore important to attain the desired separating properties.

In general, titania membranes are prepared from sol-gel methods and there are several strategies pursued by the research community to tailor the pore size of titania membranes. Ayral and co-workers developed mesoporous titania membranes by controlling the sintering temperature of the titania nanocrystal phase [10] and by using triblock copolymer templates [11]. In another work, it was reported that inhibiting the growth of particles by using organic additives was beneficial for the molecular weight (MW) cut-off of 820 Da [12]. Tsuru and co-workers [13] tested their titania membranes in several salts of sodium, magnesium chlorides, and sulphates and found good MW control between 500 and 1000 Da, as well as reported the effect of surface charge on the rejection of salts. There is also a number of conventional parameters in the sol-gel method that can be used to control the pore size of final membranes including pH, water-to-titanium ratio, and synthesis temperature, in addition to membrane calcination conditions such as ramping rates and temperatures.

The majority of the titania membranes are prepared as thin films coated on porous substrates. This creates an asymmetric structure, with the thin film having smaller pore sizes while the substrate with the larger pore sizes provides the desired mechanical strength for the membrane. The sol-gel method is a versatile route for the synthesis of thin films on substrates, generally by dip-coating where the substrate is immersed in a titania solution. The latter adheres to the surface of the substrate upon withdrawal from the solution. Upon calcination, the final structural properties of the membranes will be influenced by the particle size of the deposited titania films [14] and the calcination temperature above 700 °C which may lead to the transformation of anatase to the rutile phase of titania films [15]. There are also other methods used to prepare titania membranes including plasma [16], atomic layer deposition [17], chemical vapour deposition (CVD) [18], and self-assembly plasma-assisted CVD [19]. A noticeable emerging method for the preparation of membranes is vacuum-assisted pore size tailoring of thin films, but it is as of now untested for the preparation of metal oxide membranes. This method has been only recently reported to provide pore size control for mixed matrix carbon alumina membranes, by infiltrating phenolic resins into alumina substrates [20] and also for carbon thin films coated on alumina substrates [21]. However, the effect of the vacuum-assisted method is not known for inorganic structures derived from metal oxides.

Therefore, this work focuses on studying the effect of the vacuum-assisted method in tailoring the pore size of titania membranes. To this end, prepared titania membranes were exposed to varying vacuum times and the resultant materials were fully characterised. Furthermore, the titania membranes were investigated for MW cut-off in various organic substances to determine the correlations between the material structure and membrane performance. The titania membranes were also tested for oil/water separation using an oil emulsion of hexadecane with a high oil concentration (3000 ppm). Finally, a mechanism is proposed to explain the effect of the vacuum-assisted method on pore size variance.

## 2. Results and Discussion

### 2.1. Materials and Membrane Characterisation

**Figure 1:**
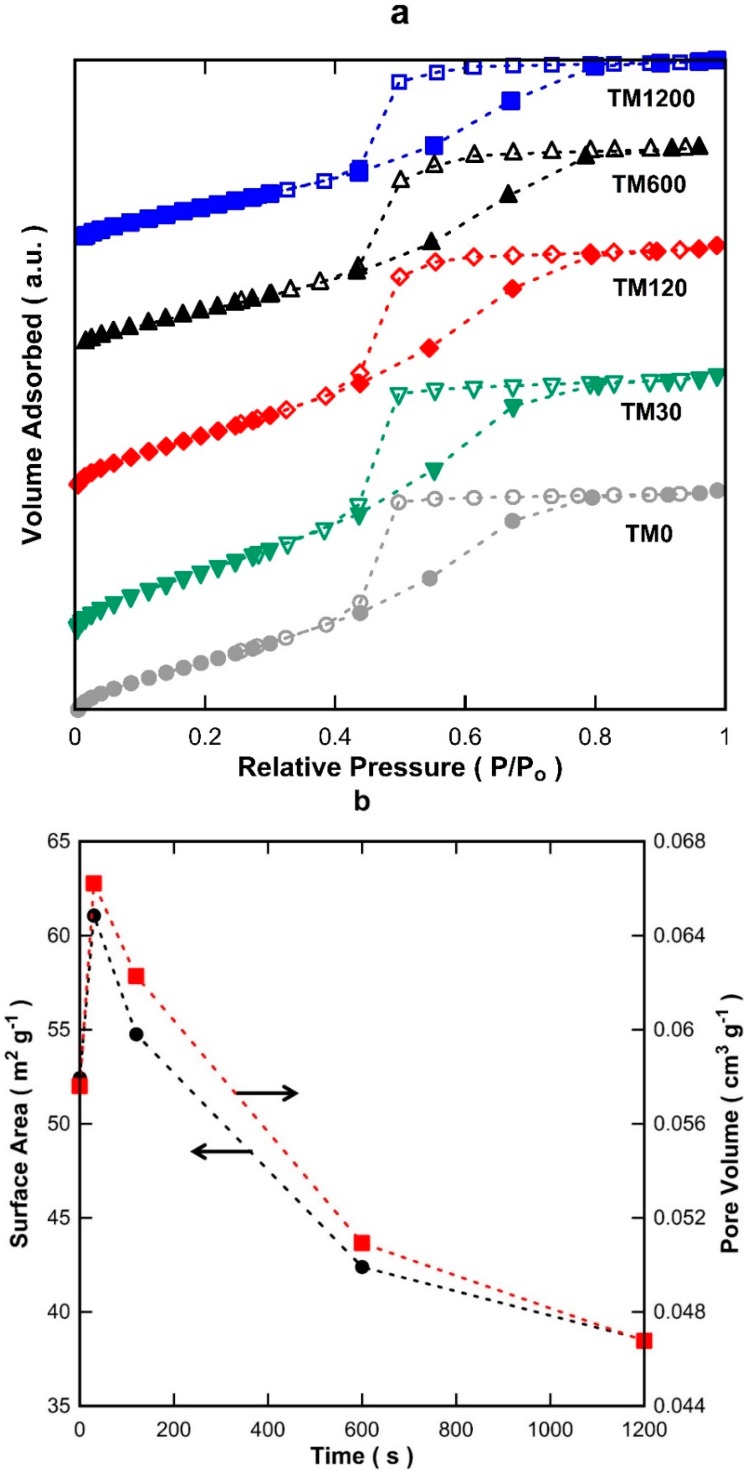

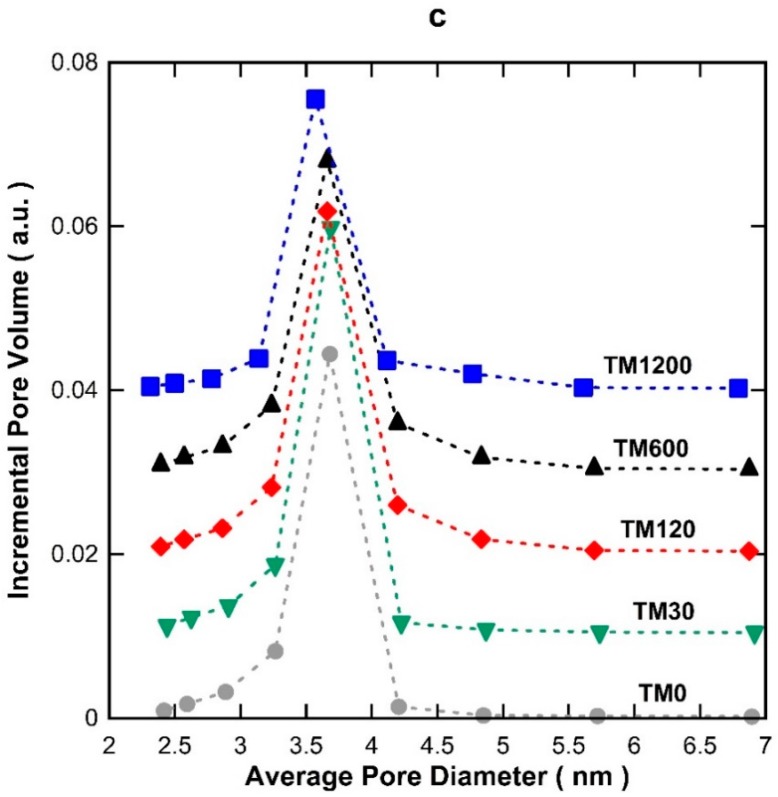
(**a**) Nitrogen adsorption isotherms (full symbols for adsorption and hollow symbols for desorption); (**b**) Brunner-Emmett-Teller (BET) surface area and pore volumes; and (**c**) pore size distribution of titania membranes (TM) xerogels exposed to varying vacuum times.

Figure 1a displays the isotherms of titania materials with 0, 30, 120, 600, and 1200 s of vacuum exposure (TM0 to TM1200). All isotherms ascribed to a typical IUPAC Type-IV profile based on the adsorption/desorption behaviour, which is the characteristic of mesoporous structures [22]. The desorption branch of the isotherms clearly illustrates hysteresis which is further indicative of a mesoporous material. The isotherms of all the TM materials appear quite similar which strongly suggests that all samples have similar textural properties and morphological features. Figure 1b shows the Brunner-Emmett-Teller (BET) surface areas and pore volumes. As can be observed, the surface areas of the TM membranes increased from TM0 to TM30. After 30 s of vacuum time, the surface areas declined gradually from 61.1 to 38.5 m^2^·g^−1^, of which the latter is for TM1200. The pore volumes also followed a similar trend, consistent with exposure to vacuum at different times. The pore size distribution (PSD) of the TM materials in Figure 1c display a single modal distribution with an average pore size of 3.6 nm.

**Figure 2:**
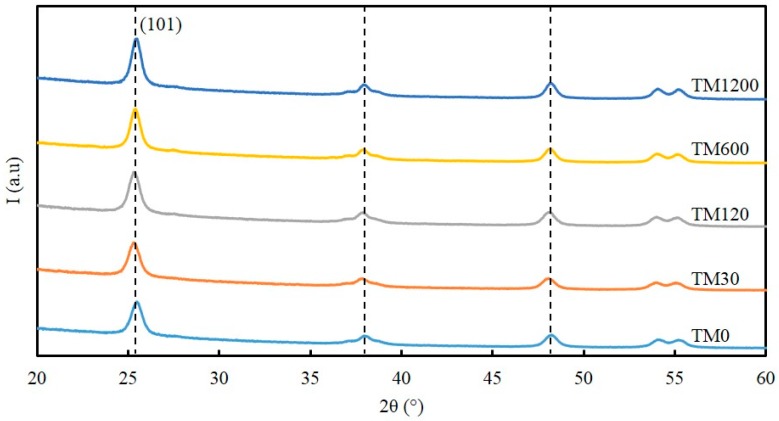
X-ray diffraction (XRD) patterns of titania samples calcined at 400 °C and exposed to different vacuum times.

The X-ray diffraction (XRD) patterns of all the calcined TM samples in Figure 2 display five sharp distinct peaks at 25.7°, 37.4°, 48.3°, 54.1°, and 55.4° 2θ, which corresponds to crystallite reflections of {101}, {004}, {200}, {105} and {211} of titania material assigned to TiO_2_-anatase (JCPDF-894121; *I*4_1_/*amd* space group symmetry) [23]. The Scherrer equation was used to calculate the average TiO_2_-anatase crystallite size based on the peak width at half maximum of the {101} reflection [24], and the results are listed in Table 1. The crystallite size of the anatase titania was calculated to be 12.2 nm for the TM0 sample but reduced to 11.5 nm for the TM30 sample. By further increasing the vacuum time, the crystallite size of the anatase titania increased systematically to a maximum value of 12.9 nm for the TM1200 sample. These features are also consistent with the BET surface area and pore volume trends observed in Figure 1b.

**Figure 3:**
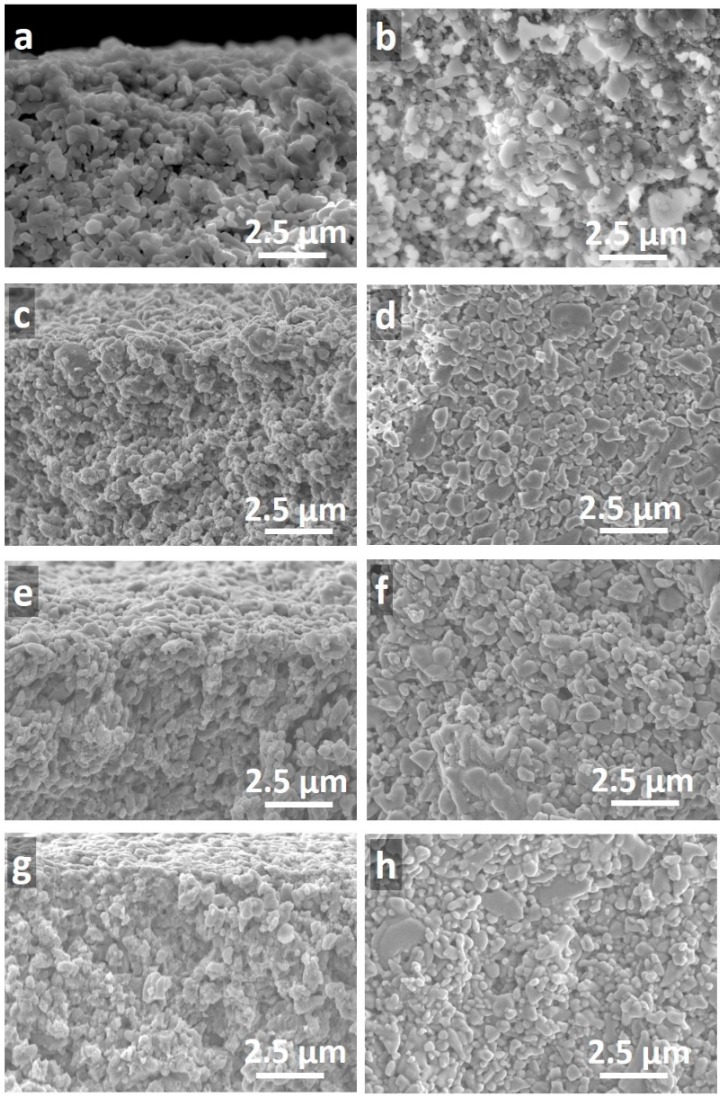
Scanning electron microscopy (SEM) of the cross-section of (**a**) alumina substrate and the TM1200 membranes for (**c**) one layer (**e**) two layers (**g**) three layers and their corresponding surface morphology of (**b**) alumina substrate (**d**) one layer (**f**) two layers and (**h**) three layers of TM1200 membranes.

Representative scanning electron microscopy (SEM) images of a blank alumina support and three titania membranes with one, two, and three layers of titania prepared at 1200 s of vacuum exposure (TM1200-1L, TM1200-2L, and TM1200-3L) are shown in Figure 3. From these images, titania thin films could not be observed on the top of any of the alumina substrates. This suggests a significant impregnation of the titania sol upon contact with the dry porous alumina substrate, and/or to the vacuum exposure of 1200 s. On closer inspection of Figure 3g,h, only the TM1200-3L membrane appeared to compose of a very light coverage of titania as a result of the multiple dip-coating and vacuum process to form a thicker and more compact film with smaller particles on the top surface.

### 2.2. Molecular Weight (MW) Cut-Off Investigations

**Figure 4:**
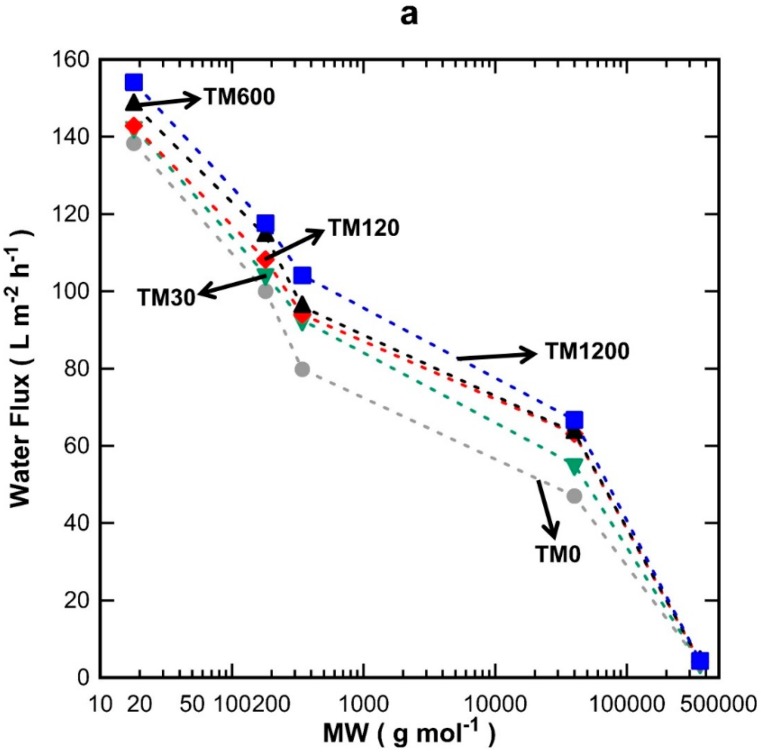

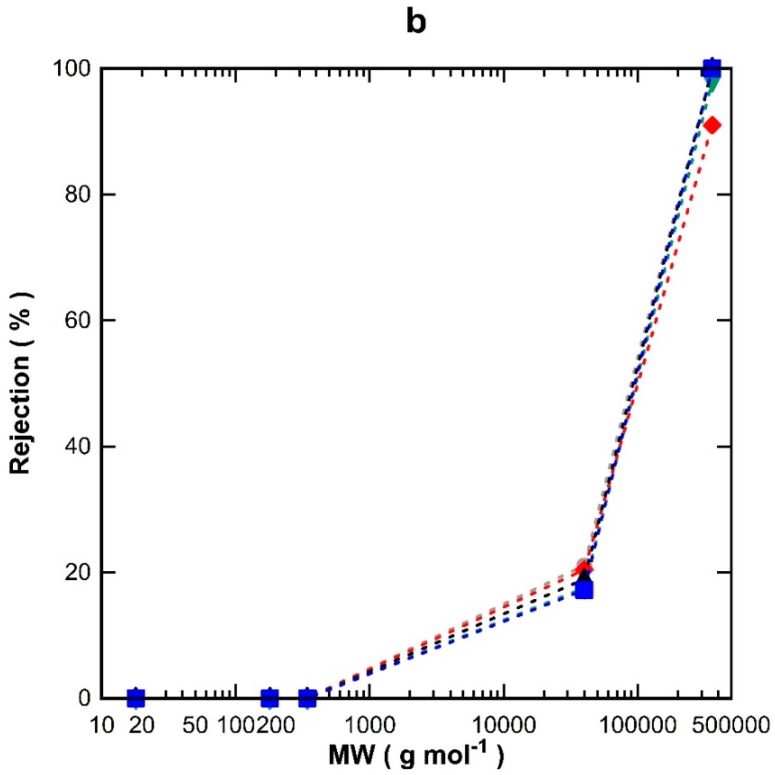
Single layer TM membranes at different vacuum times (**a**) flux and (**b**) percentage of rejection as a function of molecular weight (MW) for TM0 (grey circles), TM30 (green inverted triangles), TM120 (red diamonds), TM600 (black triangles) and TM1200 (blue squares).

MW cut-off testing was carried out on TM1 membranes (one layer) as a function of vacuum exposure time, and on TM1 to TM3 membranes (exposed to a fixed 1200 s vacuum) with one to three layers of titania. Figure 4a shows the discreet water flux for all the TM1 membranes tested using water, glucose, sucrose, PVP-40 kDa, and PVP-360 kDa molecules. It can be seen that water flux increased proportionally with vacuum time and decreased with increasing the MW of the substances tested. In Figure 4b, it is observed that the single layer TM1 membranes were not able to separate glucose (0.86 nm) [25] and sucrose (0.9 nm) [26], thus demonstrating percolation pathways with pore sizes larger than 0.9 nm. The rejection values increased to ~17% for PVP-40 kDa and to values close to 100% for PVP-360 kDa. A minor variation is observed for the rejection of PVP-360 kDa for TM1-120 which did not quite reach 100%, unlike the other TM1 membranes prepared with different vacuum times. This could be attributed to minor film defects and/or the variation in the morphology of the substrates, due to the cheap, low-quality alumina substrates employed in this work as a means to reduce fabrication cost.

**Figure 5:**
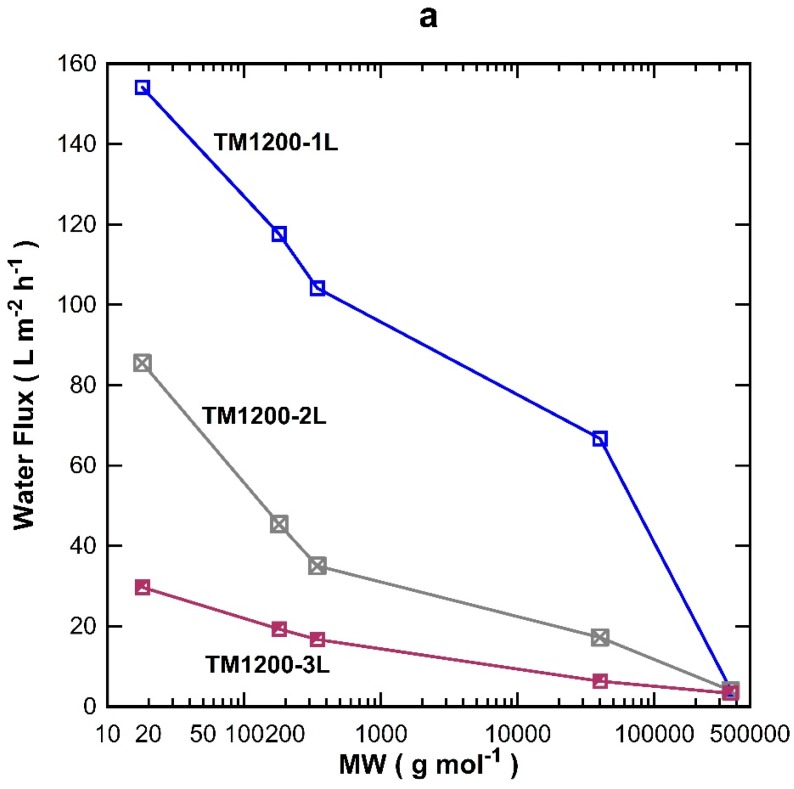

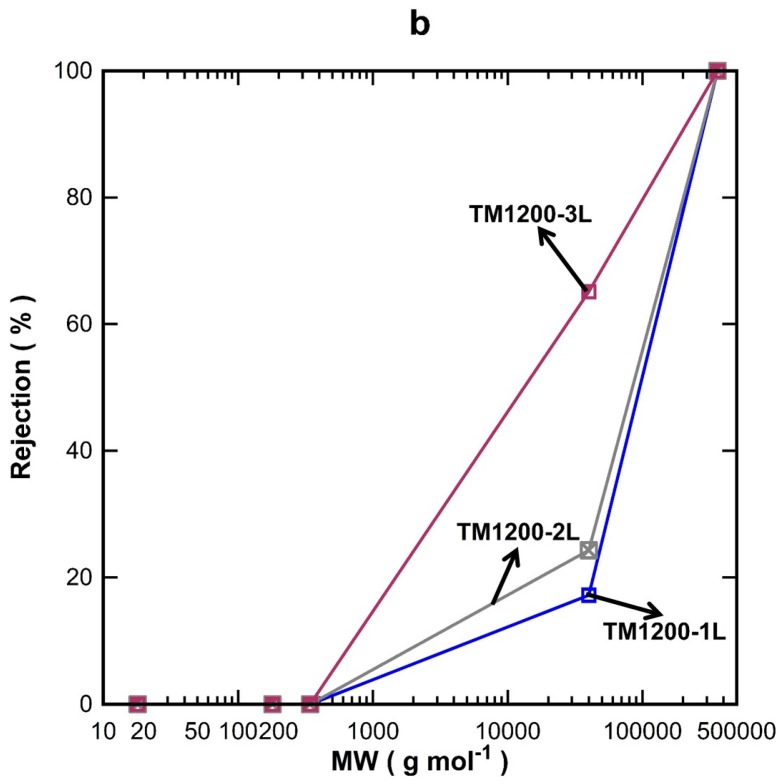
MW cut off performance of TM1200 membranes prepared with varying numbers of titania layers (**a**) flux and (**b**) percentage of rejection as a function of MW.

The MW cut-off testing was also carried out on the titania membranes prepared with one, two, and three coated-layers exposed for 1200 s of vacuum. Overall, the water flux permeation in Figure 5a decreased with increasing the MW of the organic substances, which is in good accord with the results for the TM1200 membrane vacuum series. TM1200-1L (single layer) gave the highest flux for all the tested organics compared to two and three layered TM membranes, of which their fluxes reduced sequentially as the number of layers increased to two and three layers. These results clearly indicate that the number of coating layers increased the mass resistance and thus decreased the flux. By plotting the rejection results as a function of the MW (Figure 5b), it was observed that the rejection increased with the number of titania coating as well as the MW of the organics, which is in concert with similar trends discussed for Figure 4b. The TM1200-1L membrane with a single layer revealed the lowest rejection of 17% for PVP-40 kDa as compared to two and three layer membranes. The TM1200-3L membrane delivered 65% rejection for PVP-40 kDa, thus suggesting the membrane pore sizes are reduced as more layers are added to the TM membranes. Indeed, it is very likely that by increasing the number of layers, the membrane pore sizes gradually narrowed, thus causing interlayer pore wall thickening. Finally, values close to 100% rejection were measured for PVP-360 kDa for all TM membranes, which demonstrates that the membrane pore sizes of these TM membranes are much smaller than the PVP-360 kDa molecules.

### 2.3. Oil and Water Separation

**Figure 6:**
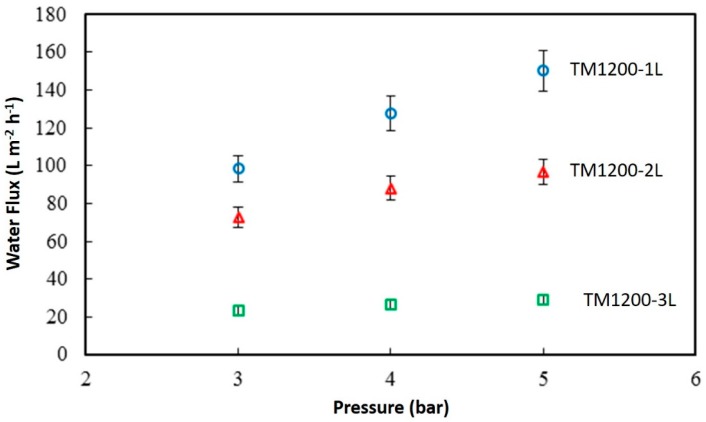
Water flux of the TM1200 membranes as a function of the feed pressure and number of layers.

The best TM membranes prepared using a vacuum time of 1200 s were further tested in pure water prior to oil-water separation. Figure 6 shows the discreet water flux of the titania membranes as a function of the feed pressure. TM1200-1L membrane with a single coat has the maximum flux of 150.4 L·m^−2^·h^−1^ at 5 bar, compared to TM1200-3L which presented the lowest flux with 29.2 L·m^−2^·h^−1^ for the same transmembrane pressure difference. Overall, the lowest water flux value was produced by TM1200-3L membrane with 23 L·m^−2^·h^−1^ at the lowest tested pressure of 3 bar. A clear trend is observed when raising the feed pressure which predictably resulted in increasing the water flux of all the membranes systematically. In addition, the flux is inversely proportional to the number of titania coatings on the substrate. For instance, the water flux at 5 bar for TM1200-1L is 36% and 81% higher than that of TM1200-2L and TM1200-3L membranes, respectively.

**Figure 7:**
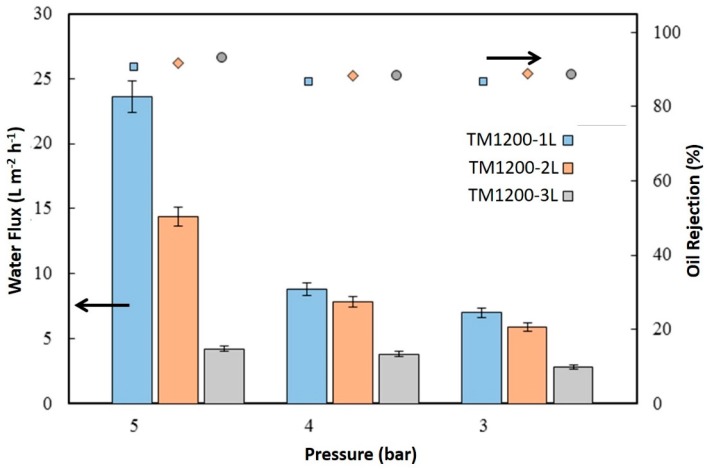
Water flux of the TM1200-1L, TM1200-2L, and TM1200-3L membranes tested with oil emulsion (3000 ppm of hexadecane) at 60 min of filtration.

Figure 7 displays the water flux of the membranes from oil-water separation testing using 3000 ppm of hexadecane feed solution. It is important to make a qualification at the outset for which the oil water permeation/separation tests were carried out initially at a feed pressure of 5 bar, then 4 bar, and then 3 bar. The membranes were also chemically cleaned after each pressure test was finished due the appearance of strong oil fouling. The highest flux by the TM1200-1L membrane reached 23.6 L·m^−2^·h^−1^ at 5 bar in an oil filtration test. Again, the TM1200-3L membrane gave the lowest flux of 2.8 L·m^−2^·h^−1^ at 3 bar. The water fluxes of all the titania membranes for oil/water separation followed the same trend as for pure water testing, where water fluxes increased as a function of the feed pressure. However, the trend in oil rejection was inversely proportional to water flux trends. For instance, the highest water flux TM1200-1L membrane delivered the lowest oil rejection of 90.7% whilst the lowest water flux TM1200-3L membrane produced the highest oil rejection of 93%.

The TM membranes showed excellent durability and reusability during the MW cut-off investigation. The TM membranes at different vacuum times were tested with five different species (i.e., water, glucose, sucrose, PVP-40 kDa, and PVP-360 kDa), where each membrane was tested at least three times for each condition, totaling 15 tests. The TM membranes always reached steady state permeation after each testing, and after each cleaning protocol with water was applied. In the case of oil/water separation, the TM membranes were tested over multiple cycles of pressure (i.e., 5, 4, and 3 bar) and again at least three times for each testing condition. After each testing point, the membranes were chemically cleaned using sodium hydroxide and citric acid. However, the cleaning process did affect the performance of the TM membranes, thus suggesting that chemical cleaning was detrimental for the long term durability of the TM membranes.

### 2.4. Discussion and Analysis of Structural Formation

In this work, cheap alumina substrates were used where the substrate pore sizes ranged from 0.5 to 1 µm in diameter. The SEM images of the TM membranes illustrate that thin films were not formed on the substrate surface for the first and the second titania layers. A slight thin film was observed for the third layer only. Therefore, the first consideration is whether the titania sol could penetrate into the pores of the alumina substrate. From Table 1, the XRD results show that the crystallite sizes of the TM materials exposed to vacuum time varied between 11.5 and 13.0 nm. However, these crystallite size measurements only represent the size of a single titania crystal, hence for the TM samples the anatase titania phase is likely to be formed by several crystals. In order to estimate the size of the titania nanoparticles, assuming that the titania particles are round, the following relationship can be used to calculate the radius of the particles:

$$r=\frac{3}{S\rho }$$

where *r* (µm) is the radius, *S* (m^2^·g^−1^) is the BET surface area determined by nitrogen adsorption, and *ρ* (g·cm^−3^) is the density. As the titania phase is mainly anatase, the density is 3.78 g·cm^−3^.

Table 2 lists the diameter of the titania particles exposed to vacuum. The particle sizes were calculated between 0.026 and 0.041 µm. These particle sizes are much smaller than the alumina substrate pore sizes (0.5 to 1 µm), and therefore can reside within the alumina substrate due to the titania sol impregnation via dip-coating and by the action of the vacuum. These results therefore explain why the titania films are not observed on the top of alumina substrates by the SEM images. In addition, it is interesting to observe that the trends in Table 2 are found to be the same as in Table 1. In other words, the particle size and crystallite size decrease from TM0 to TM30, and then both sizes increase thereon as a function of the vacuum time.

**Figure 8:**
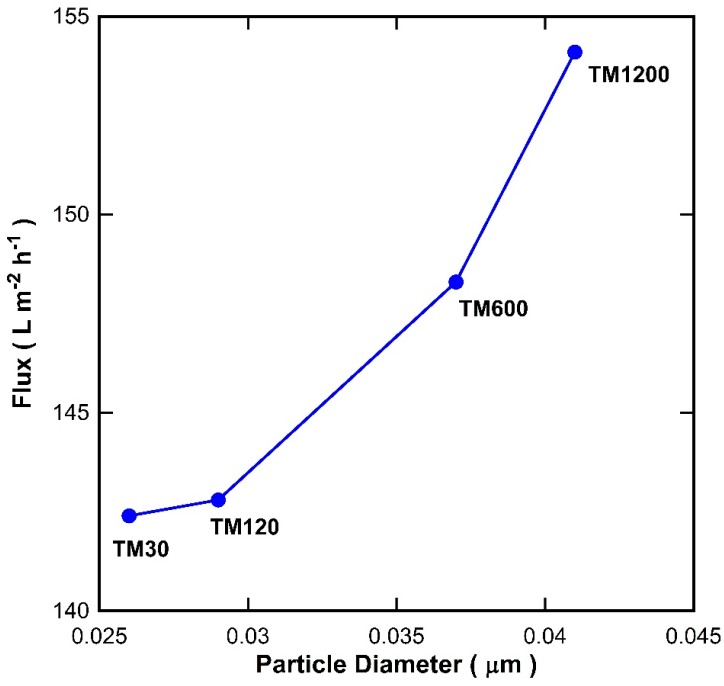
Water flux for single layer titania membranes versus calculated titania particle diameter.

It is well known that the void between the particles increases as the particle size increases. To examine this theory, the particle size in Table 2 were plotted against the pure water flux for the TM membranes with a single titania coating exposed to different vacuum times as shown in Figure 8. It is clearly observed that there was a good fit between the particle size and the water flux. As the particle size of titania increases, the water flux of the TM membranes increases. These results therefore strongly suggest that the vacuum time played an important role in changing the particle size of the titania, which in turn alters the inter-particle voids and thus affects the membrane resistance to water transport across the membranes.

To understand these relationships more in depth, the titania particles formed via the sol-gel process using titania isopropoxide as a precursor should be discussed. The chemistry of the sol-gel method is mainly based on hydrolysis shown in Equation (1) and polycondensation reactions shown in Equations (2) and (3) of metal alkoxides, leading to the formation of an extended network [27].

Ti–OR + H_2_O **→** Ti–OH + ROH (1)

Ti–OH + OR–Ti **→** Ti–O–Ti + ROH (2)

Ti–OH + HO–Ti **→** Ti–O–Ti + H_2_O (3)

**Figure 9:**
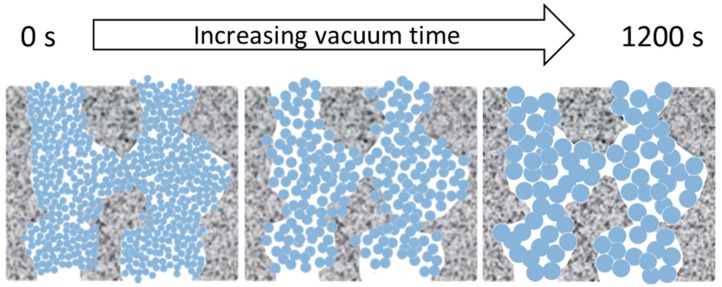
Schematic of the effect of the vacuum in the formation of titania films.

The morphological features and MW cut-off results of the TM materials and membranes as a function of vacuum times is interesting. Therefore, we propose that the morphological features of the TM material are due to a combined effect of the sol-gel reactions and vacuum, leading to Ostwald ripening of the titania crystals and nanoparticle growth from the initial sol-gel network to the anatase titania matrix in a sequential manner, as schematically depicted in Figure 9 and described as follows:

(1) By applying a vacuum, the longer the vacuum time, the more likely that the Ti–O–Ti networks are brought in closer proximity to each other, which will form small titania nuclei upon which the other Ti precursors can be grown.
(2) As Ti–O–Ti network formation proceeded as a function of the vacuum time, the aggregation of the networks slightly increased and caused an increased crystallite size upon calcination, accompanied by an increase in particle size.
(3) The Ti–O–Ti networks formed small particles inside the porous alumina substrate to create well packed films.
(4) The increase in particle size likewise increased the inter-particle void.

The mechanism postulated for the TM membranes as a fucntion of the vacuum time is supported by the SEM images which show well packed films. As the N_2_ adsorption results showed a single, narrow pore size distribution centering at approximately 4 nm, these results strongly suggest that the calcination process led to the formation of titania particles. This is further supported by the XRD patterns of the titania particles with only the anatase phase morphology. Therefore, as the particle size enlarges, the inter-particle void becomes larger which further supports the increase of water flux as the vacuum time is increased.

## 3. Experimental

### 3.1. Materials and Characterisation

The titania sol was synthesised based on a previously reported method [2]. Briefly, the hydrolysis and condensation of titanium tetrapropoxide (TTP, Ti(OC_3_H_7_)_4_, 97%) was carried out in acidic conditions using hydrochloric acid. TTP, water, and HCl were mixed at a molar ratio of 1:23.2:0.9 and the sol was then aged under stirring at room temperature for 2.5 h. Subsequently, the sol was poured onto large petri dishes and placed inside a desiccator where a vacuum was applied for the desired time. After drying, the titania xerogels were calcined at 150 °C followed by a second calcination at 400 °C. For both stages of calcination in air, the heating and cooling rate of 1 °C·min^−1^ was used with a dwell time of 1 h.

Structural properties of the titania xerogel were analysed using an ASAP 2020 apparatus (Micromeritics Instrument Corporation, Norcross, GA, USA). The samples were degassed at 200 °C for 24 h under high vacuum prior to nitrogen adsorption at 77 K. Thermogravimetric analysis (TGA) and differential scanning calorimetry (DSC) of the xerogels were studied using Mettler-Toledo TGA/DSC 1 (Mettler-Toledo, Columbus, OH, USA) from 30 to 400 °C at the same calcination conditions as the prepared titania xerogels. The crystalline phases of the titania xerogels were detected via X-ray diffraction (XRD) which was conducted using a Bruker D8 Advance (Bruker, Billerica, MA, USA). XRD patterns were collected at 40 kV and 20 mA with graphite monochromators using filtered Cu Kα radiation. The tested range of 2θ was from 30° to 90°.

### 3.2. Membrane Preparation, Characterisation, and Testing

TM membranes were prepared by coating the outer shell of alumina tubular substrates (Ceramic Oxide Fabricators, Melbourne, Australia) with titania sols. The alumina tubes of ~5 cm in length and ~1 cm in outer diameter were initially pre-calcined at 1000 °C prior to dip-coating. The external surface of the alumina was exposed to the diluted titania sol by a dip-coating method, with a holding time of 1 min and withdrawal rate of 10 cm·min^−1^. Subsequently, the coated tube was immediately exposed to a vacuum pressure (<1 Torr), where the vacuum was applied via the inner shell of the tube for a desired time ranging from a short time of 30 s to a longer time of 1200 s. Upon vacuum exposure, the membranes were calcined in air using the same calcination condition as the titania xerogels above. The cycle of dip-coating, vacuum and calcination procedure was repeated for membranes coated with 2 and 3 layers.

**Figure 10:**
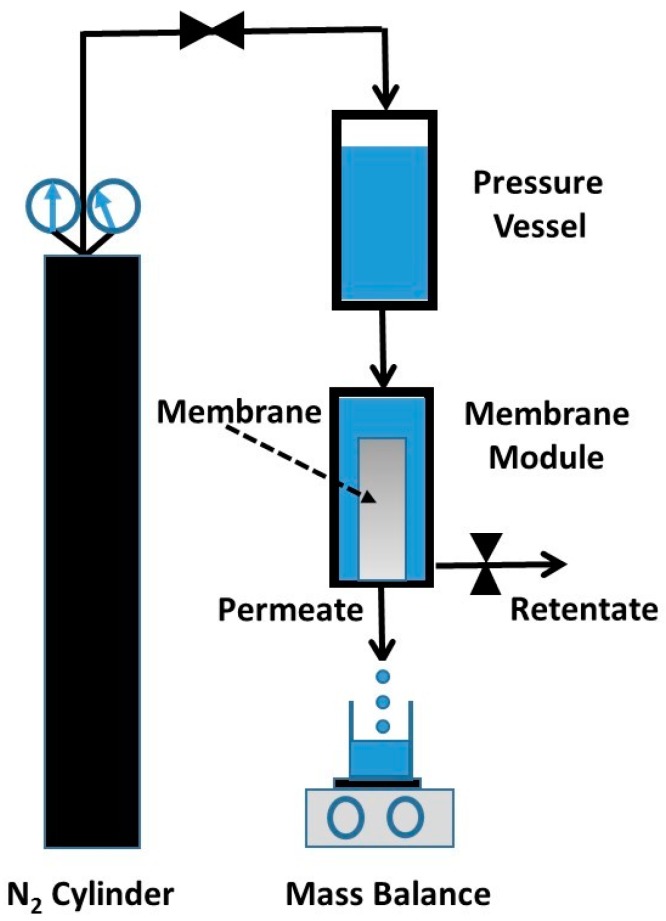
Schematic of the membrane experimental set up.

The titania membranes were tested for water permeation and the rejection of organic substances, and oil/water separation, using a dead-end (i.e., closed retentate stream) permeation rig, as schematically shown in Figure 10. The aqueous solution column in pressure vessel was pressurised using nitrogen gas to desired pressures. This set up emulates the effect of pumps, so the pressure of the feed side is higher than that of the permeate side, thus providing the driving force (i.e., pressure gradient) required for the permeation of water through the membrane. The permeate was collected in a beaker on an electronic scale linked to a computer, so the permeate mass could be determined as a function of time to calculate the water flux as *J* = ∆*W/A*∆*t*, where *J* (L·m^−2^·h^−1^) is the water flux, ∆*W* (kg) is the permeated mass collected over a predetermined time ∆*t* (h) at steady state, and *A* (m^2^) is the titania membrane area.

Molecular weight (MW) cut-off studies were carried out in aqueous solutions containing 3000 ppm concentrations of glucose, sucrose, and polyvinyl pyrrolidine (PVP molecular weights of 36 kDa and 400 kDa). The samples collected from the permeate stream were analysed using a Shimadzu UV-2700 UV-Vis spectrometer (Shimadzu Corporation, Kyoto, Japan) to determine their concentration against calibrated curves. The rejection (*R*) of substances was calculated using the following equation: *R* = [1 − (*C_p_*/*C_f_*)] × 100, where *R* (%) is the rejection, and *C_p_* and *C_f_* are the concentrations of the samples collected from the permeate and feed streams, respectively.

## 4. Conclusions

Titania membranes (TM) prepared using different vacuum exposure times (30 to 1200 s) were investigated by molecular weight cut-off and oil emulsion filtration along with their material characterizations. A strong trend was found in that the water flux of the membranes decreased with the increasing molecular weight of the tested substances using water, glucose, sucrose, PVP-40 kDa, and PVP-360 kDa as a direct result of nanofiltration. Furthermore, all the membranes rejected almost 100% of the larger PVP-360 kDa molecules, albeit the membranes were not able to separate the smaller molecular sizes of glucose and sucrose, which indicates that the membrane pore sizes are larger than the kinetic diameter of sucrose of 0.9 nm. The TM membranes were able to separate a highly concentrated oil emulsion (3000 ppm) delivering rejections from 90% to 93%, which increased from a single layer to three layers of membrane coating, respectively, although water fluxes trended oppositely due to increasing mass transfer resistance through the membranes. Characterisation of materials revealed that the structural formation of the TM membranes was affected by the vacuum time which promoted aggregation of the Ti–O–Ti networks, leading to the creation of increased anatase phase TiO_2_ nanoparticles as the vacuum exposure time increased. Likewise, this mechanism led to larger inter-particle voids with a larger total pore volume, thus reducing the mass transfer resistance as membrane pore sizes increased as a function of the vacuum time.

## Figures and Tables

**Table 1:** The crystallite size of titania material based on X-ray diffraction (XRD) patterns in Figure 2.

Sample	Crystallite Size (nm)
TM0	12.2
TM30	11.5
TM120	11.8
TM600	12.9
TM1200	12.9

**Table 2:** Calculated titania particle sizes.

TM Material	Particle Diameter (µm)
TM0	0.030
TM30	0.026
TM120	0.029
TM600	0.037
TM1200	0.041
